# Investigating Specific Associations Between Childhood Victimization Profiles and Positive Psychosis Symptoms: The Mediating Roles of Anxiety, Depression, and Schema

**DOI:** 10.1093/schizbullopen/sgad017

**Published:** 2023-06-15

**Authors:** Georgina L Barnes, Richard Emsley, Philippa Garety, Amy Hardy

**Affiliations:** Department of Psychology, Institute of Psychiatry, Psychology and Neuroscience, King’s College London, London, UK; South London and Maudsley NHS Foundation Trust, Bethlem Royal Hospital, London, UK; Department of Biostatistics and Health Informatics, Institute of Psychiatry, Psychology and Neuroscience, King’s College London, London, UK; Department of Psychology, Institute of Psychiatry, Psychology and Neuroscience, King’s College London, London, UK; South London and Maudsley NHS Foundation Trust, Bethlem Royal Hospital, London, UK; Department of Psychology, Institute of Psychiatry, Psychology and Neuroscience, King’s College London, London, UK; South London and Maudsley NHS Foundation Trust, Bethlem Royal Hospital, London, UK

**Keywords:** childhood trauma, poly-victimization, psychosis, delusions, anxiety

## Abstract

**Background:**

Childhood trauma is a risk factor for psychosis. It is proposed this is due to traumatic events giving rise to psychological mechanisms that are implicated in the development and maintenance of symptoms. Investigation of the psychological mechanisms accounting for relationships between trauma and psychosis will be assisted by focusing on specific trauma profiles, hallucination modalities, and delusion subtypes.

**Study Design:**

In 171 adults with schizophrenia-spectrum diagnoses and high-conviction delusions, associations between childhood trauma classes, and hallucination and delusion factors, were tested using structural equation models (SEMs). Anxiety, depression, and negative schema were examined as potential mediators of trauma class-psychosis symptom factor links.

**Study Results:**

Significant associations were found between the emotional abuse/neglect and poly-victimization classes with persecutory delusions and delusions of influence, that were all mediated through anxiety (β = 1.24–0.23, *P* = < .05). There was an association between the physical abuse class and grandiose/religious delusions that was not explained by the mediators (β = 1.86, *P* = < .05). Trauma class was not significantly associated with any hallucination modality (β = 0.004–1.46, *P* = > .05).

**Conclusions:**

In a sample of people with strongly held delusions, this study demonstrates that childhood victimization is associated with delusions of influence and grandiose beliefs, as well as with persecutory delusions in psychosis. Consistent with previous findings, the potent, mediating role of anxiety supports affective pathway theories and the utility of targeting threat-related processes when treating trauma effects in psychosis.

## Introduction

Interacting biopsychosocial factors are implicated in the etiology and maintenance of psychosis, and include exposure to childhood victimization. Research has consistently shown strong associations between childhood trauma and psychosis severity, persistence, and diagnoses in case-control and cohort designs.^1–4^ There is also evidence that poly-victimization may have an additive risk effect and that the resolution of trauma is associated with a reduction in psychosis.^5–7^ These findings have led to claims that trauma plays a causal role in psychosis, at least for a subgroup of people.^8,9^ This is of clinical significance, as trauma in psychosis is associated with reduced antipsychotic effectiveness and worse clinical and functional outcomes.^10,11^

Identification of biopsychosocial mechanisms underlying trauma and psychosis would provide further support for causality and highlight potential targets for intervention. Hardy^12,13^ proposes a potential role for trauma-related emotions, negative schematic beliefs, episodic memories, and emotional regulation in psychosis, building on seminal cognitive-behavioral theories.^14–16^ There is meta-analytic evidence for the mechanisms proposed in Hardy’s model, including trauma-related emotions, beliefs, re-experiencing, avoidance, hyperarousal, dissociation, and attachment, although study quality is often low, and larger samples and robust designs are needed.^17–19^

A key debate in the area is whether childhood victimization has a generic impact on mental health difficulties and underlying biopsychosocial mechanisms, or if there is a degree of specificity between trauma types and outcomes, such as psychosis. There is emerging evidence that childhood trauma types may impact certain psychosis symptoms through distinct cognitive-affective processes.^20,21^ For example, a specific association has been found between sexual abuse and auditory hallucinations^22,23^ which may be particularly driven by threat-related anxiety processes, such as dissociation or hyperarousal.^12,24^ For example, shutting down and hypervigilance are understandable, automatic responses to sexual abuse, which may contribute to hallucinations due to their impact on sensory-perceptual processing, with ambiguous sounds becoming more salient and perceived as threatening voices. A specific relationship between emotional abuse and neglect with persecutory delusions has also been identified,^22,25,26^ which is hypothesized to be fueled by the impact of trauma on negative schema about the self and others.^17,27^ For instance, verbal bullying may lead to negative beliefs about harm from others, giving rise to anxiety, and threat-related social processing. This could increase vulnerability to persecutory appraisals of ambiguous social experiences, such as interpreting someone’s facial expression as a sign of negative judgement. These studies demonstrate the significance of trauma in psychosis and suggest that specific subtypes of trauma may have differential effects on psychosis symptoms through trauma-related emotions and negative schematic beliefs. However, a consensus has not been reached about the role of affect and schema as mediators in specific trauma-psychosis relationships, and further research is required in this area.

A limitation of existing research in this field is that co-occurring trauma types are often not explored and specific psychosis symptom factors are not typically investigated. Studies of trauma in psychosis have tended to report global symptom categories (ie, positive vs negative) and/or symptom types (eg, hallucinations vs delusions). For example, Bailey et al^28^ conducted a meta-analysis and identified that emotional abuse and neglect were specifically linked to hallucinations, although the picture for delusions was more equivocal, with neglect not reaching significance and a modest relationship found with sexual abuse. However, this level of psychosis specificity may not be sufficiently precise. It would be informative to investigate hallucination modalities or delusion subtypes, given that distinct processes may play a role in the phenomenology of different types of positive symptoms; eg, persecutory vs grandiose beliefs. In line with this, a recent study has investigated relationships between trauma clusters and voice hearing dimensions and found poly-victimization was associated with more negative content and clinically significant voice hearing.^29^ This suggests trauma severity, rather than type, may be most influential in determining the content and impact of voices. However, to our knowledge, no study has examined hallucination modalities and delusional subtypes in relation to co-occurring trauma types. Understanding these links may help to refine psychological models and tailor therapeutic approaches for people with psychosis.

In a retrospective analysis using data from 2 randomized experimental studies,^30,31^ we aimed to investigate specific trauma-psychosis associations by exploring relationships between childhood trauma classes and factors of hallucination modalities and delusion subtypes. Anxiety, depression, and negative schema were explored as potential mediating mechanisms in identified links between traumatic events and specific psychosis outcomes, in line with existing cognitive-behavioral theories of trauma in psychosis.^12–16^

## Methods

### Participants

This study was a retrospective analysis of 2 wider randomized experimental studies investigating cognitive mechanisms in persecutory delusions and paranoia.^30,31^ Inclusion criteria for both studies were: aged between 18 and 65 years with a schizophrenia-spectrum diagnosis (nonaffective, ICD-10 F20–F28) and a current delusion held with at least 50% conviction and persisting for at least 3 months, assessed on the Schedules for Clinical Assessment in Neuropsychiatry (SCAN).^32^ Both studies recruited participants from community psychosis teams in the UK. In total, 171 participants meeting the inclusion criteria and who completed the key assessment measures (see below) were included in the current study.

### Procedure

The original studies from which the current sample was taken^30,31^ were reviewed by an NHS research ethics committee, and all participants provided written informed consent. Demographic data and psychosis symptom measures were collected at baseline as part of the wider experimental studies. Trauma measures and assessment of psychological processes were collected at a 3-month follow-up; however, this was a cross-sectional study.

#### Trauma Assessment.

*The Trauma History Questionnaire* (THQ)^33^ is a structured interview that assesses lifetime trauma exposure. For this study, items relating to Childhood Sexual Abuse (CSA), Childhood Emotional Abuse (CEA), and Childhood Physical Abuse (CPA) types were used. The THQ has acceptable psychometric properties in psychosis samples.^34^

*The Childhood Experience of Care and Abuse Questionnaire* (CECA-Q)^35^ is a self-report measure assessing caregiving experiences prior to aged 17 years. For this study, we used the Antipathy and Neglect subscales. The CECA-Q has well-established validity and reliability.^36^

#### Psychosis: Hallucination Modalities and Delusion Subtypes.

*The Scales for the Assessment of Positive Symptoms* (SAPS)^37^ is a clinician-administered tool that contains 35 items measuring positive psychosis symptoms over the past month. Items are rated on a 6-point scale (0–5), with higher ratings indicating greater symptom severity. For this study, items used were: *Hallucinations* (6 items: 1. Auditory; 2. Voices Commenting; 3. Voices Conversing; 4. Tactile/Somatic; 5. Olfactory; and 6. Visual); *Delusions* (12 items: 1. Persecutory; 2. Jealousy; 3. Guilt/Sin; 4. Grandiose; 5. Religious; 6. Somatic; 7. Reference; 8. Being controlled; 9. Mind being read; 10. Thought broadcast; 11. Thought Insertion; and 12. Thought withdrawal). The SAPS has good psychometric properties.^38^

### Psychological Mediators

*The Beck Depression Inventory* (BDI)^39^ and *Beck Anxiety Inventory* (BAI)^40^ are 21-item self-report questionnaires assessing symptoms of depression and anxiety over the past 2 weeks. For both measures, items are rated on a 4-point scale (0–3) providing a total score (0–63), with higher scores reflecting greater depression/anxiety severity. The BAI and BDI have robust psychometric properties.^41,42^

*The Brief Core Schema Scale* (BCSS)^43^ is a 24-item self-report questionnaire assessing negative and positive beliefs about the self and others. There are 4 subscales: Negative Self/Other and Positive Self/Other. Each item is rated on a 5-point scale (0–4), with higher scores indicating greater belief strength. The scale has good psychometric properties.^43^

#### Data Analysis.

All analyses were performed using Stata version 15.0.^44^ First, binary variables were created for the presence or absence (0 = no trauma, 1 = trauma) of childhood trauma across 4 variables: Sexual abuse (THQ); Physical abuse (THQ); Emotional abuse (THQ and CECA-Q Parental Antipathy); and Neglect (CECA-Q Parental Neglect). The method for capturing the 4 trauma indicators across the THQ and CECA-Q in this sample has previously been described.^45^

Latent class analyses (LCAs) were performed on available data from 146 participants reporting at least 1 type of childhood trauma using Maximum Likelihood estimation as described previously.^45^ Four distinct childhood trauma classes were identified in the sample: emotional abuse/neglect (*n* = 29); physical abuse (*n* = 14); sexual abuse (*n* = 19); and poly-victimization (*n* = 84). The classes were compared to a no childhood trauma group (*n* = 20), which was treated as the reference category in the mediation analyses.

Two exploratory factor analyses (EFAs) were then conducted on the SAPS hallucinations and delusions items, respectively. The ordinal score (0–5) for each item was used. Factors were rotated using an oblique (Oblimin) procedure with Kaiser Normalization, to provide information on correlations amongst the factors.

Finally, structural equation models (SEMs) were fitted to explore associations between the 4 trauma classes and psychosis symptom factors. Linear regressions were employed to test associations between trauma class and the 4 mediators (anxiety, depression, negative self, and negative other schemas). For significant trauma class × mediator associations, SEMs were estimated to investigate trauma class-psychosis symptom links; 1 model for hallucinations and 1 for delusions, both conditional on the no trauma group and any significant mediator(s) identified at stage 1. Causal mediation analysis was then performed using the product of coefficients method.

Guidelines have been published for determining the sample size required for mediational studies with 80% statistical power.^46^ This is based on evidence of the minimum sample size needed to identify a mediated effect using SEM software (*n* = 152).^47^ We estimated that a sample *n* = 171 would have 80% power to detect a significant effect of trauma class on the primary outcome of psychosis symptoms and the associated mediators.

## Results

### Participants

The total sample of 171 participants had a mean age of 42.2 years (SD = 10.9, range = 19–65). The majority were male (*n* = 106; 62%) and 58.5% (*n* = 100) identified as White British/Other. Most participants had an ICD-10 diagnosis of Paranoid Schizophrenia (*n* = 150; 88%), and the remaining had diagnoses of Schizoaffective Disorder (*n* = 10; 6%), Delusional Disorder (*n* = 8; 5%), or Other Nonorganic Psychosis (*n* = 2; 1%). All demographic information is presented in table 1.

**Table 1.** Demographic and Clinical Characteristics of the Sample (*n* = 171)

Variable	Mean or *n*	SD or %
Age		
Mean (SD)	42.2	(10.9)
Gender		
Male	106	(62.0)
Female	65	(38.0)
Ethnicity		
White British/ Other	100	(58.5)
Black British/African/Caribbean	45	(25.3)
Asian British/Indian/Pakistani	8	(4.7)
Mixed British/Mixed Other	14	(8.2)
Other ethnicity	4	(2.3)
English first language		
Yes	154	(90.1)
No	17	(9.9)
Civil status		
Single	121	(70.8)
Married or cohabiting	16	(9.4)
Separated	33	(19.3)
Other	1	(0.6)
Employed		
Yes	15	(8.8)
No	156	(91.2)
Clinical measures		
SAPS hallucinations	2.8 (0–5)	(1.7)
SAPS delusions	4.1 (3–5)	(0.6)
Depression (BDI total)	25.2 (1–58)	(12.7)
Anxiety (BAI total)	22.1 (0–62)	(12.8)
Negative Self Schema (BCSS-Self)	7.7 (0–23)	(5.9)
Negative Other Schema (BCSS-Other)	11.2 (0–24)	(6.9)

*Note*: *M*, mean; *n*, number of cases.

### Rates of Childhood Victimization in the Sample

For the 146 participants who reported at least 1 type of childhood trauma, the most frequently endorsed victimization experiences were emotional bullying (50.3%), unwanted sexual experiences (35.5%), and physical abuse within the home (34.7%). Less commonly reported trauma types were forced sexual intercourse (16.8%), assault with a weapon (18.0%), and maternal neglect (19.2%). Descriptive data on childhood trauma rates in this sample have previously been described.^45^

### Factor Structure of Hallucination Modalities

The final factor analysis loaded the hallucination items onto 2 clear and interpretable factors. The rotated factor loadings for all SAPS hallucinations items are reported in table 2. The oblimin rotation method was deemed acceptable as the 2 factors were strongly correlated (*r* = 0.52). The first factor represented auditory hallucinations (3 items), which included items related to voices and other auditory experiences, which accounted for 86.1% of the variance; the second factor represented multimodal hallucinations (3 items), which included items related to visual, olfactory and tactile phenomena and accounted for 13.9% of the variance. Hallucination severity scores for the 2 factors were derived by combining the scores on each item making up the auditory and multimodal hallucinations factors.

**Table 2.** Rotated Factor Loadings for SAPS Hallucination Items

Item	Factor 1: Auditory Hallucinations	Factor 2: Multimodal Hallucinations	Uniqueness
1. Auditory hallucinations	**0.81**	0.01	0.33
2. Voices commenting	**0.79**	0.09	0.29
3. Voices conversing	**0.77**	−0.10	0.48
4. Visual hallucinations	0.03	**0.53**	0.84
5. Olfactory hallucinations	−0.01	**0.45**	0.83
6. Tactile/somatic	0.07	**0.41**	0.78

Significant associations are in bold.

### Factor Structure of Delusion Subtypes

An initial EFA of the items extracted only 1 factor with an eigenvalue > 1, so a 3-factor solution was run. Factor loadings with an absolute value <0.32 were suppressed, as were items that cross-loaded highly onto other factors. As the somatic item loaded weakly onto all 3 factors, it was excluded from the analysis. Correlations were low between all factors (>0.3) so the analysis was re-run using an orthogonal rotation.

The final factor analysis loaded 10 delusions items onto 3 distinct, clinically interpretable factors. The rotated factor loadings for all SAPS delusions items are reported in table 3. Orthogonal rotation was deemed an acceptable method as the 3 factors were weakly correlated (*r* = 0.1–0.5). The first factor represented delusions of influence (6 items) which included items related to passivity phenomena (eg, thought broadcast/withdrawal) and referential beliefs and accounted for 71.3% of the variance. The second factor represented grandiose delusions with religiosity (2 items) which included items relating to grandiose/religious ideas and accounted for 15.0% of the variance. The third factor represented persecutory delusions (2 items) which included items related to beliefs of persecution and guilt/sin and accounted for 13.7% of the variance. Delusion severity scores for the 3 factors were created by combining the scores on each item making up the delusions of influence, grandiose/religious, and persecutory delusions factors.

**Table 3.** Rotated Factor Loadings for SAPS Delusions Items

Item	Factor 1: Delusions of Influence	Factor 2: Grandiose/religious Delusions	Factor 3: Persecutory Delusions	Uniqueness
1. Persecutory	0.07	0.05	**0.43**	0.82
3. Guilt or sin	−0.03	0.09	**0.41**	0.82
4. Grandiose	0.04	**0.56**	0.00	0.68
5. Religious	0.13	**0.57**	0.13	0.66
7. Reference	**0.34**	0.24	0.19	0.76
8. Controlled	**0.61**	0.08	0.12	0.59
9. Mind read	**0.49**	0.21	0.11	0.70
10. Broadcast	**0.53**	0.05	−0.10	0.70
11.Insertion	**0.56**	0.04	−0.13	0.67
12. Withdrawal	**0.55**	0.04	−0.01	0.69

Significant associations are in bold.

### Linear Regressions of Trauma Class-Mediator Associations

Significant associations were found between the emotional abuse/neglect (β = 0.23, *P* = .03) and poly-victimization (β = 0.28, *P* = .02) classes with anxiety severity. There was no significant effect of trauma class on depression (β values = −0.05 to 0.16, *P* values > .1), negative self-schema (β values = −0.03 to 0.14, *P* values >.05), or negative other schema (β values = −0.16 to 0.18, *P* values > .1), therefore anxiety was the only mediating variable entered in the final mediation models.

### Mediation Model for Hallucination Modalities

There was a significant effect of anxiety on the severity of auditory hallucinations (β = 0.11, *P* = .001), and multi-modal hallucinations (β = 0.10, *P* = .001). Trauma class did not predict auditory hallucination severity (β values = 0.28–1.46, *P* > 0.05) or multi-modal hallucinations (β values = 0.004–1.26, *P* > .05), when anxiety was controlled for, indicating mediation. When anxiety was included in the final model, there was no effect of trauma class on auditory or multi-modal hallucinations (β values = 0.32–0.80, *P* > .05). The final model was found to have a poor fit (χ^2^: 14.12, *P* = .00; RMSEA: 0.279). Table 4 displays the direct, indirect, and total effects of trauma class on the 2 hallucinations factors.

**Table 4.** The Effect of Trauma Class on Emotions and Schema (Path a)

Trauma class	Emotions		Schema	
	Anxiety β (SE)	Depression β (SE)	Negative Self β (SE)	Negative other β (SE)
Class 1: Emotional abuse/neglect	**0.24** ^*^ **(0.04)**	0.16 (0.03)	0.15 (0.03)	0.17 (0.05)
Class 2: Physical abuse	0.13 (0.02)	-0.09 (0.04)	−0.04 (0.02)	0.16 (0.05)
Class 3: Sexual abuse	0.13 (0.02)	0.05 (0.03	−0.04 (0.02)	0.17 (0.04)
Class 4: Poly-victimisation	**0.28** ^*^ **(0.05)**	0.16 (0.03)	0.09 (0.03)	0.18 (0.03)

*Note:* Standardized Beta coefficients from ordinary least-squares linear regressions, conditional on no trauma group. Gender included as covariate in all regressions.

^*^Significance: *P* < .05.

Significant associations are in bold.

### Mediation Model for Delusion Subtypes

There was a significant effect of anxiety on persecutory delusions (β = 0.33, *P* = .001) and delusions of influence (β = 0.16, *P* = .000), but not grandiose delusions with religiosity (β = 0.01, *P* = .43). Trauma class did not predict persecutory delusion severity (β values = −0.58 to 0.54, *P* > .05) or delusions of influence (β values = −0.40 to 1.86, *P* > 0.05), when anxiety was controlled for in the model, indicating mediation. When controlling for anxiety, physical abuse (β = 1.80, *P* = .02) and poly-victimization (β = 1.06, *P* = .04) predicted the severity of grandiose/religious delusions, suggesting specific trauma-symptom associations that were not mediated through anxiety.

When anxiety was included in the final model, there was a significant effect of emotional abuse/neglect on delusions of influence (β = 1.24, *P* = .05), and with poly-victimization on persecutory delusions (β = 0.23, *P* = .05), indicating that these relationships were mediated fully through anxiety. There was also an effect of poly-victimization on delusions of influence (β = 1.11, *P* = .04) which did not account for the total effect, indicating that anxiety partially mediated this relationship. The final model had an adequate fit (χ^2^: 7.89, *P* = .048; RMSEA: 0.098). Table 5 shows the direct, indirect, and total effects of trauma class on the 3 delusions factors.

**Table 5.** Direct, Indirect and Total Effects of Trauma Class on Hallucinations, With Anxiety as the Mediating Variable

Trauma Class	Symptom	Path *c*ʹ (Direct) β (SE), *P*	Path *ab* ­(Indirect) β (SE), *P*	Path *c* (Total) β (SE), *P*
Emotional abuse and neglect	Auditory hallucinations	−0.42 (1.43), .77	0.80 (0.45), .07	0.05 (0.25), .84
	Multi-modal hallucinations	−0.22 (0.79), .79	0.45 (0.25), .07	0.09 (0.17), .62
Physical abuse	Auditory hallucinations	1.46 (1.79), .41	0.65 (0.50), .19	0.36 (0.31), .25
	Multi-modal hallucinations	0.004 (0.98), .99	0.36 (0.28), .20	0.14 (0.22), .63
Sexual abuse	Auditory hallucinations	−1.21 (1.57), .44	0.58 (0.45), .20	−0.08 (0.27), .78
	Multi-modal hallucinations	−1.26 (0.86), .14	0.32 (0.25), .19	−0.18 (0.19), .35
Multiple abuse	Auditory hallucinations	0.28 (1.23), .82	0.72 (0.39), .06	0.14 (0.21), .50
	Multi-modal hallucinations	0.33 (0.68), .63	0.40 (0.22), .06	0.17 (0.15), .26

*Note*: β = Standardized β coefficient from structural equation models; conditional on the no trauma group and controlling for sex.

## Discussion

To the best of our knowledge, this is the first study to show evidence of links between latent classes of childhood trauma and specific delusion subtypes in a sample of adults with strongly held delusional beliefs in psychosis. Using stringent tests of trauma-psychosis associations, we found evidence for specific associations from trauma classes to delusions of influence, persecutory, and grandiose delusions. The focus on delusions of influence and grandiosity was novel, as prior research examining the specificity of trauma-delusion associations has tended to only focus on persecutory or referential beliefs. The identification of theoretically driven psychological mechanisms underlying specific relationships between childhood victimization and delusion subtypes provides further support for the role of trauma in psychosis (table 6).

**Table 6.** Direct, Indirect and Total Effects of Trauma Class on Delusions, With Anxiety as the Mediating Variable

Trauma class	Symptom	Path cʹ (direct) β (SE), *P*	Path ab ­(indirect) β (SE), *P*	Path c (total) β (SE), *P*
Emotional abuse and neglect	Persecutory delusions	0.17 (0.45), .71	0.26 (0.14), .07	0.42 (0.46), .36
	Grandiose/religious	0.99 (0.62), .11	0.09 (0.11), .45	1.08 (0.62), .08
	Delusions of influence	0.96 (1.48), .52	1.24 (0.63), .05*	2.20 (1.57), .16
Physical abuse	Persecutory delusions	−0.58 (0.56), .30	0.18 (0.15), .25	−0.40 (0.57), .49
	Grandiose/religious	1.80 (0.78), .02*	0.06 (0.89), .50	1.86 (0.77), .02*
	Delusions of influence	−0.40 (1.83), .83	0.86 (0.73), .24	0.46 (1.95), .81
Sexual abuse	Persecutory delusions	0.54 (0.49), .27	0.18 (0.14), .20	0.72 (0.51), .15
	Grandiose/religious	0.33 (0.68), .63	0.06 (0.09), .49	0.39 (0.68), .57
	Delusions of influence	0.61 (1.63), .71	0.90 (0.66), .18	1.50 (1.73), .39
Multiple abuse	Persecutory delusions	0.17 (0.39), .66	0.23 (0.12), .05*	0.40 (0.39), .31
	Grandiose/religious	1.06 (0.54), .04*	0.08 (0.10), .45	1.14 (0.53), .03*
	Delusions of influence	1.86 (1.26), .14	1.11 (0.55), .04*	2.98 (1.34), .03*

*Note*: Standardized β coefficient from structural equation models; conditional on the no trauma group and controlling for sex.

Significant associations are in bold.

*P < 0.05.

A specific association was found between poly-victimization and delusions of influence, mediated partly through anxiety, but not depression or negative schema. This links to previous research which has identified the role of affective processes underlying relationships between childhood victimization and passivity phenomena in psychosis.^48^ For example, dissociation is an anxiety-driven threat response and there is a well-established relationship between dissociation and psychosis.^7^ Anxiety and dissociation in response to poly-victimization may impact people’s sense of agency over mental events, such that internal experiences are perceived as being externally influenced.^18^ These mechanisms may also contribute to external events having heightened salience for an individual, leading to referential ideas.^49^ As anxiety partly accounted for this relationship, this means that there are likely to be other cognitive-affective mechanisms driving the relationship from poly-victimization to passivity experiences and referential beliefs that require further investigation.

We further identified a specific relationship between childhood physical abuse and poly-victimization with grandiose/religious delusions, indicating that grandiose beliefs in psychosis are relevant in trauma. There was no evidence of mediation through anxiety, depression, or negative schema. Grandiose delusions have been found to be partially explained by the extent to which they provide a sense of coherence, purpose, and significance in a person’s life, which may be in response to adversity and trauma.^50,51^ Therefore, different types of psychological mechanisms may play a role in grandiosity to those investigated in this study, particularly when they represent a protective response to past trauma and its consequences. In terms of specific processes, grandiose delusions have previously been associated with appraisals of anomalous experiences^52^ and maintained by cognitive reasoning biases and repetitive imagery-based thinking.^53,54^ This highlights the need for more focused empirical research on grandiose delusions and their meaning for people with psychosis and trauma histories.

We also found significant associations between emotional abuse/neglect and persecutory delusions, which have been identified in previous empirical studies.^23,25,26^ These findings were not consistent with the meta-analytic findings from Bailey et al,^28^ which identified that only sexual abuse and total trauma, not neglect, was associated with delusions. This suggests that assessment of co-occurring trauma types and delusion subtypes may be more sensitive when identifying trauma-psychosis links. In addition, anxiety was found to be a significant mediator of this relationship, which supports the role of threat-related processes in persecutory delusions.^55^ This finding aligns with the view that psychological threats in interpersonal relationships may play a particularly potent role in paranoia.^21^ In terms of other psychological mechanisms, negative schema did not mediate the relationship between emotional abuse/neglect and paranoia, as has been found in other empirical studies.^17,20,21^ It is possible that this may be attributable to sampling biases, with ceiling effects due to the severity of participants’ beliefs. Therefore, in the future, it will be useful to test these trauma-symptom links using similar approaches in other clinical groups.

Lastly, we did not find a specific relationship between CSA and auditory hallucinations, as has been found in previous work.^21,23,28^ We did find some evidence for a relationship between poly-victimization and auditory and multi-modal hallucinations, which was only present when anxiety was included in the model; however, this did not reach statistical significance. This may, in part, be due to sample characteristics, as this sample was selected specifically for the presence of high-conviction delusions. Nonetheless, the association between anxiety and hallucinations aligns with existing theories of an affective, threat-processing pathway to psychosis.^12,56–58^ The finding that anxiety-mediated trauma-delusion subtype links is also consistent with recent meta-analyses that support anxiety as a trauma-related mechanism that contributes to positive symptoms.^17–19^ The mediating role of anxiety is also consistent with a network analysis of post-traumatic stress and psychosis symptoms which found that hypervigilance was central to the relationships between post-traumatic stress symptoms and delusions.^59^

### Limitations

**Fig. 1.**
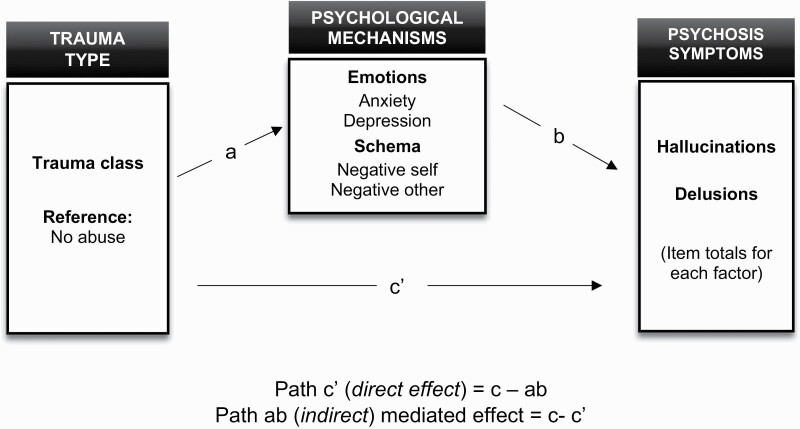
The proposed mediation framework for effects of trauma class on hallucinations and delusions.

Study limitations should be considered. In our analysis, we used hallucination modalities and delusion subtypes to investigate trauma-psychosis symptom links. This addresses the limitations of previous research which has investigated global symptom categories (ie, positive vs negative) and/or global symptom types (eg, hallucinations vs delusions). However, this study did not explore the phenomenological features of hallucinations and delusions, which may be a more precise method for capturing the content of psychosis symptoms. This is important given that recent research has found specific links between symptom phenomenology and childhood trauma in psychosis, such as voices reflecting negative self-content and impact.^29,60–62^ Further research may therefore seek to explore patterns of symptom characteristics (eg, negative content, degree of controllability, symptom-related distress) across childhood trauma subtypes to advance understanding of the relationship between types of traumatic experiences and clinically important characteristics of hallucinations and delusions (figure 1).

Another limitation is that we collapsed individual trauma items into 4 categories and did not analyse them individually. We decided these 4 categories a priori as the number of trauma classes was unknown due to this being an exploratory study. However, this may have obscured specific trauma profiles. Future research investigating victimization profiles in psychosis could include a broader range of trauma types and include measures that capture different dimensions of trauma, such as environment (inside vs outside the home) and event type (single vs ongoing).

Another limitation is that the lack of significant indirect effects between trauma class and psychosis symptom factors could have been due to the small number of participants assigned to certain classes which could have reduced statistical power. In addition, our mediation models indicated that trauma class caused changes in anxiety and, in turn, anxiety caused changes in hallucinations and delusions. However, as the data were collected at 1 time point, causal ordering cannot be determined. Further research using longitudinal designs may help to investigate causal claims about the mediation of specific trauma-psychosis relationships. As a retrospective analysis, we did not investigate key potential mediators implicated in the trauma-psychosis relationship, such as emotional regulation and memory processes.^12,13^ A final limitation is that some recruitment data from the wider trials were unavailable for the current study; including the number of subjects excluded by the 3 criteria and data on the number of people invited to participate who declined or did not respond. As a result, we were unable to explore potential important differences between groups.

Our findings underscore the importance of recognizing and assessing trauma and its consequences in people with psychosis, given that this may play a role in symptom development and maintenance. This has important implications for implementing trauma-informed models of care in services for people with psychosis^63^ and the need to comprehensively yet concisely assess trauma in this population.^64^ Mental health practitioners working in clinical services, therefore, require support and training to increase awareness of the prevalence and consequences of childhood trauma in psychosis and use routine enquiry to assess different types of abuse and neglect. This may help to further guide trauma-informed support for those accessing services.

The current study also highlights the potent, mediating role of anxiety in associations between trauma types and psychosis symptoms. This supports affective pathway theories and the clinical utility of targeting threat-related processes in psychological interventions for people with psychosis and a history of trauma. This could include a combination of emotion regulation skills, cognitive restructuring, and exposure-based techniques. Rigorous evaluations of these interventions are currently being conducted as part of the STAR and Re.Process randomized controlled trials^65,66^ which will help to improve our understanding of the dynamic associations between trauma-related processes and psychosis outcomes.
